# Thermally Fine-Tuned NiO_x_–MAPbI_3_ Interfaces Enabled by a Polymeric Surface Additive for High-Sensitivity Self-Powered Photodetectors

**DOI:** 10.3390/polym18030375

**Published:** 2026-01-30

**Authors:** HyeRyun Jeong, Kimin Lee, Wonsun Kim, Byoungchoo Park

**Affiliations:** Department of Electrical and Biological Physics, Kwangwoon University, Wolgye-Dong, Seoul 01897, Republic of Korea; skad10004@naver.com (H.J.); dlrlals1123@naver.com (K.L.); dnjsun21@naver.com (W.K.)

**Keywords:** polymeric surfactant, interfacial engineering, noise-limited detectivity, self-powered photodetector, perovskite photodiodes, nickel oxide (NiO_x_) hole-transport layer

## Abstract

Self-powered perovskite photodiodes provide an attractive platform for low-power and high-sensitivity photodetection; however, their performance capabilities are often constrained by inefficient interfacial charge extraction and noise suppression. Here, we report a polymer-mediated interfacial engineering strategy for methylammonium lead iodide (MAPbI_3_) photodiodes by integrating thermally optimized nickel oxide (NiO_x_) hole-transport layers (HTLs) with a nonionic polymeric surfactant, poly(oxyethylene)(10) tridecyl ether (PTE). NiO_x_ films annealed at 300 °C establish a favorable energetic baseline for hole extraction, while the ppm-level incorporation of PTE into the MAPbI_3_ precursor enables the molecular-scale modulation of the NiO_x_/MAPbI_3_ interface without forming an additional interlayer. The external quantum efficiency at 640 nm increases from 78.7% for pristine MAPbI_3_ to 84.1% and 84.6% for devices incorporating 30 and 60 ppm PTE, corresponding to enhanced responsivities of 406, 434, and 437 mA/W. These improvements translate into reduced noise-equivalent power and an increase in the noise-limited detectivity from 2.50 × 10^12^ to 2.76 × 10^12^ Jones under zero-bias operation. Importantly, enhanced sensitivity is achieved without compromising the dynamic performance, as all devices retain fast temporal responses and kilohertz-level bandwidths. These results establish polymeric-surfactant-assisted interfacial engineering as a scalable and effective platform for low-noise, high-sensitivity self-powered perovskite photodiodes for renewable-energy-integrated systems.

## 1. Introduction

Self-powered photodetectors that operate without external bias are highly attractive for next-generation optoelectronic systems, as they offer intrinsically low power consumption, reduced circuit complexity, and enhanced operational stability [1,2,3,4]. Among the various emerging materials, hybrid organic-inorganic perovskites have garnered considerable attention with regard to photodetection owing to their strong optical absorption, long carrier diffusion lengths, ambipolar transport capability, and solution processability [5,6,7,8,9]. In particular, methylammonium lead iodide (MAPbI_3_) has been widely explored for self-powered photodiodes, enabling high responsivity, a broad linear dynamic range (*LDR*), and rapid responses under zero-bias operation [7,8,9,10,11,12]. These attributes make MAPbI_3_-based photodiodes promising for weak-light sensing, imaging, and low-power optoelectronic applications.

Despite these advantages, the performance of self-powered MAPbI_3_ photodiodes is still fundamentally limited by inefficient interfacial charge extraction and noise-related loss mechanisms. Under zero-bias operation, photocarrier separation relies entirely on the built-in electric field, leading to devices that are particularly sensitive to interfacial recombinations, energetic barriers, and defect-assisted noise generation [8,13,14,15,16]. Consequently, optimizing the interface between the perovskite absorber and charge-transport layers is a critical requirement for achieving high detectivity and reliable weak-light detection.

Nickel oxide (NiO_x_) has emerged as a leading hole-transport layer (HTL) for perovskite optoelectronics due to its wide bandgap, high optical transparency, deep valence-band position, and superior chemical stability compared to polymeric HTLs such as poly(3,4-ethylenedioxythiophene) polystyrene sulfonate (PEDOT:PSS) [12,17,18,19,20]. In contrast to polymeric HTLs such as PEDOT:PSS, which is hygroscopic and acidic and is widely reported to induce moisture ingress, interfacial corrosion, ion migration, and accelerated degradation in perovskite devices, NiO_x_ is an inorganic p-type oxide that exhibits negligible hygroscopicity, strong resistance to electrochemical degradation, and favorable interfacial compatibility with perovskite absorbers [18]. These intrinsic advantages of these materials enable NiO_x_-based interfaces to maintain stable energy-level alignment and low electronic noise under prolonged self-powered operation, making NiO_x_ a more reliable platform for low-power and stability-critical perovskite optoelectronic applications. Moreover, solution-processed NiO_x_ is compatible with scalable fabrication routes. However, the electronic structure, stoichiometry, and surface morphology of NiO_x_ are highly sensitive to the processing conditions, particularly thermal annealing [21,22,23,24,25]. Variations in the annealing temperature can significantly alter the Ni^3+^/Ni^2+^ ratio, work function (WF), and surface roughness, thereby influencing the energy-level alignment and interfacial recombinations at the NiO_x_/MAPbI_3_ interface [21,25,26,27]. While thermal optimization of NiO_x_ has been shown to improve the perovskite photovoltaic performance, its implications with regard to noise characteristics, detectivity, and dynamic responses in self-powered photodiodes remain insufficiently understood.

To improve the interfacial charge extraction further, various interfacial engineering strategies have been explored, including the insertion of ultrathin interlayers, chemical doping, and surface passivation [14,28,29,30,31,32]. Although effective, these approaches often introduce additional processing complexity, increase the device thickness, or risk perturbing the bulk properties of the perovskite layer. In this context, molecular-based interfacial modifiers offer an attractive alternative for tailoring interfacial energetics without introducing additional structural complexity. In particular, nonionic polymeric surfactants possess unique advantages arising from their amphiphilic molecular structures. These characteristics enable the molecular-level tuning of the interfacial energetics and morphology without the formation of discrete interlayers and without compromising the bulk crystallinity [33,34,35,36,37]. Despite their widespread use in perovskite film formation, the role of polymeric surfactants as deliberate electronic modifiers at perovskite/oxide interfaces has not been systematically explored in self-powered photodetectors. Unlike small-molecule surfactants or ionic additives, polymeric surfactants provide extended chain conformations and enhanced dipole stability while suppressing long-range diffusion, thereby enabling robust, spatially confined, and electronically stable interfacial modulation when combined with energetically stable inorganic HTLs such as NiO_x_.

In contrast to conventional interfacial engineering strategies that rely on the insertion of additional passivation layers, self-assembled monolayers, ionic interlayers, or polymer buffer films, a ppm-level polymeric surfactant incorporated directly into the perovskite precursor offers a markedly simpler and more scalable route to interfacial control. This additive-based approach eliminates the need for extra deposition steps, interlayer thickness control strategies, and mitigates the parasitic resistance associated with discrete interlayers, while still enabling strong and stable electronic modulation at the NiO_x_/MAPbI_3_ interface. Because the polymeric surfactant is introduced at the molecular level rather than as a separate layer, this strategy remains fully compatible with solution processing and large-area fabrication, making it particularly attractive for scalable self-powered photodetectors and low-power optoelectronic systems.

Here, we report a polymer-mediated interfacial engineering strategy for self-powered MAPbI_3_ photodiodes that is achieved by integrating thermally optimized NiO_x_ HTLs with the ppm-level incorporation of a nonionic polymeric surfactant, poly(oxyethylene)(10) tridecyl ether (PTE), into a perovskite precursor. NiO_x_ films annealed at 300 °C establish a favorable energetic baseline for efficient hole extraction, while the controlled incorporation of PTE enables the molecular-scale modulation of the NiO_x_/MAPbI_3_ interface during the perovskite film formation step. Unlike conventional interlayers, this polymeric surfactant modifies the interfacial energetics through stable dipole formation and defect interaction without perturbing the intrinsic optical absorption, crystal structure, or charge-transport pathways of MAPbI_3_. Importantly, because PTE is introduced at the ppm level directly into the perovskite precursor and NiO_x_ is prepared via a solution-processed sol–gel route, the entire device stack is inherently compatible with scalable coating techniques such as spin-coating, slot-die coating, and inkjet printing. Unlike graphene- or nanowire-based heterojunction photodetectors that rely on material transfer, nanofabrication, or epitaxial growth processes, this polymer-assisted interfacial strategy enables molecular-scale electronic modulation without additional discrete interlayers or lithography-intensive steps. Consequently, this approach is naturally suited for large-area deposition, flexible substrates, and CMOS-relevant back-end-of-line (BEOL) photodetector integration, where low thermal budgets and process simplicity are essential [38]. From a broader perspective, such self-powered perovskite photodiodes operating without external bias are highly relevant for low-power optoelectronics in renewable-energy-aware systems, where autonomous operation and minimal power consumption are essential.

Through a comprehensive combination of morphological, spectroscopic, and device-level analyses, we demonstrate that polymer-assisted interfacial dipole engineering simultaneously enhances charge extraction, suppresses electronic noise, and preserves fast temporal responses under zero-bias operation. These results establish a general and scalable framework for polymer-enabled interfacial control in perovskite photodiodes, providing new design principles for low-noise, high-sensitivity self-powered optoelectronic devices.

## 2. Materials and Methods

### 2.1. Materials

Indium tin oxide (ITO)-coated glass substrates (sheet resistance ~15 Ω/sq) were used here as transparent conducting electrodes. Nickel(II) nitrate hexahydrate (Ni(NO_3_)_2_·6H_2_O, Sigma-Aldrich, St. Louis, MO, USA), isopropyl alcohol (IPA), and ethylene glycol (EG) (Duksan, Ansan, Republic of Korea) used for the sol–gel synthesis of NiO_x_. Lead(II) iodide (PbI_2_, 99.99%) was obtained from Thermo Fisher Scientific (Waltham, MA, USA) and methylammonium iodide (MAI, >99.99%) was purchased from Greatcell Solar (Queanbeyan, Australia).

The nonionic polymeric surfactant PTE was obtained from Tokyo Chemical Industry Co., Ltd. (TCI, Tokyo, Japan) and was used without further purification. PTE possesses an average molecular weight of approximately 500 g/mol, placing it in the oligomeric-to-polymeric regime and justifying its classification as a nonionic polymeric surfactant. This molecular characteristic enables chain-mediated interfacial stabilization effects, rather than small-molecule-like behavior. For the electron-transport and cathode-modifying layers, phenyl-C_61_-butyric acid methyl ester (PCBM, >99.5%, Nano-C, Westwood, MA, USA) and bathocuproine (BCP, >95.0%, TCI, Tokyo, Japan) were employed. Anhydrous chlorobenzene (CB), dimethylformamide (DMF), and dimethyl sulfoxide (DMSO) were purchased from Sigma-Aldrich (St. Louis, MO, USA). All materials and solvents were used as received unless otherwise noted.

### 2.2. Fabrication of NiO_x_ HTLs

The ITO substrates were sequentially cleaned by ultrasonication in ethanol, a detergent solution, and deionized water for 30 min each, followed by a UV–ozone treatment lasting 5 min to improve the surface wettability. The sol–gel NiO_x_ precursor solution was prepared by dissolving nickel nitrate hexahydrate (29 mg) in a mixture of IPA and EG at a volume ratio of 2:3. The solution was stirred at room temperature for 3 h.

The NiO_x_ precursor was spin-coated onto cleaned ITO substrates at 2700 rpm for 40 s and subsequently dried at 70 °C for 1 min to remove any residual solvent. The films were then thermally annealed in air at 300 °C for 1 h to form NiO_x_ HTLs. This annealing temperature was selected based on extensive prior studies demonstrating that a temperature of approximately 300 °C yields a Ni^3+^-rich p-type NiO_x_ surface with a low defect density, high hole mobility, and a stable WF, thereby providing an energetically well-defined and reproducible baseline interface for perovskite optoelectronic devices [26]. By fixing the NiO_x_ annealing condition at this optimized baseline, the present work isolates the impact of polymer-mediated interfacial modulation without convolution from NiO_x_ thermal-treatment effects.

### 2.3. Fabrication of MAPbI_3_ Active Layers with PTE Surfactant Incorporation

MAPbI_3_ precursor solutions were prepared by dissolving PbI_2_ and MAI in a mixed solvent of DMF/DMSO (*v*/*v* = 4:1) with a total concentration of 1.0 M. For polymer-assisted interfacial engineering, PTE was directly added to the MAPbI_3_ precursor solution at concentrations of 30 or 60 ppm relative to the perovskite solids. The solutions were stirred at 70 °C for at least 3 h to ensure the homogeneous dispersion of the polymeric surfactant.

The MAPbI_3_ films were deposited onto the thermally optimized NiO_x_ HTLs by a one-step spin-coating process inside a nitrogen-filled glovebox. The precursor solution was spin-coated at 3700 rpm for 30 s, during which CB was dynamically dropped as an antisolvent 10 s before the end of spinning to promote rapid crystallization [39]. The films were then annealed at 100 °C for 20 min to complete perovskite crystallization.

The incorporation of PTE during film formation is expected to influence both the bulk crystallization behavior and interfacial interactions at the NiO_x_/MAPbI_3_ interface. Owing to its amphiphilic molecular structure, PTE can interact with oxide surface sites while remaining partially embedded within the perovskite matrix, thereby enabling molecular-scale interfacial modulation without forming a discrete interlayer [36,37,40,41].

Following the perovskite deposition step, PCBM (20 mg/mL in CB) was spin-coated at 1800 rpm for 60 s as the electron-transport layer (ETL), with this followed by the deposition of a thin BCP layer (0.5 mg/mL in IPA). Finally, Ag electrodes (120 nm) were thermally evaporated under a high vacuum (<5 × 10^–6^ Torr) through a shadow mask, defining an active device area of 0.06 cm^2^.

### 2.4. Structural, Optical, and Electrical Characterizations

The surface morphologies of the MAPbI_3_ perovskite films deposited onto the NiO_x_ HTLs were examined by top-view scanning electron microscopy (SEM; Nova Nano SEM 200, FEI, Hillsboro, OR, USA) operated at an accelerating voltage of 10 kV, with ImageJ software (version 1.54g) subsequently used to quantify the grain-size distributions. Optical absorption spectra were recorded using a UV–visible (UV–vis) spectrophotometer (8453, Agilent Technologies, Santa Clara, CA, USA) in the wavelength range of 300–900 nm. The crystalline structures of the perovskite films were analyzed by X-ray diffraction (XRD) using a Rigaku D/Max 2200 diffractometer (Rigaku Corporation, Tokyo, Japan) equipped with Cu Kα radiation (*λ* = 1.5406 Å).

Ultraviolet photoelectron spectroscopy (UPS) measurements were taken using a Nexsa X-ray photoelectron spectrometer (Thermo Fisher Scientific, Waltham, MA, USA) equipped with a He I excitation source (21.22 eV) to determine the WFs and valence-band maximum (VBM) positions of the NiO_x_ and MAPbI_3_ films. The secondary electron cutoff and valence-band regions were analyzed to evaluate the interfacial energy-level alignment.

Current–voltage (*I*–*V*) characteristics were measured using a source meter (Keithley 2400 or 2636, Tektronix Inc., Beaverton, OR, USA) under dark and illuminated conditions. Monochromatic illumination at 637 nm was provided by a calibrated laser diode (COMPACT-100G-637-A, World Star Tech, Markham, ON, Canada), and the incident optical power was controlled using neutral-density filters. External quantum efficiency (*EQE*) spectra were obtained using an incident photon-to-current efficiency (IPCE) measurement system (IQE-200, Newport, Irvine, CA, USA) calibrated with a reference silicon photodiode (IQE-SAMPLE-SI, Newport, Irvine, CA, USA).

Noise current spectral densities were evaluated under zero-bias conditions using a Keithley 2636 source meter by recording dark-current time traces and analyzing them via the fast Fourier transform (*FFT*) with a bandwidth of 1 Hz. Temporal response characteristics were measured using a square-wave-modulated laser source and a digital oscilloscope (MDO3024, Tektronix Inc., Beaverton, OR, USA), from which rise times (10–90%), decay times (90–10%), and frequency-dependent response behaviors were extracted. All measurements were taken at room temperature under zero-bias conditions using devices fabricated on NiO_x_ HTLs annealed at 300 °C.

## 3. Results and Discussion

### 3.1. Formation of MAPbI_3_ Active Layers with PTE Surfactant Incorporation on NiO_x_ HTLs

**Figure 1:**
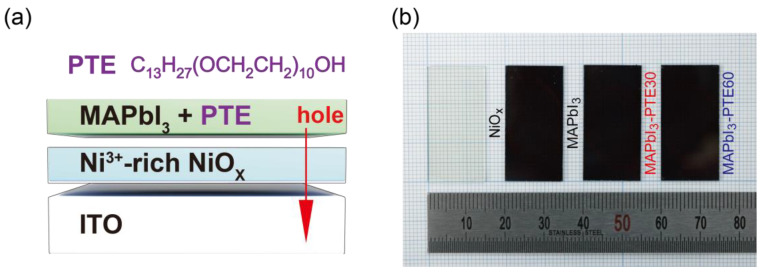
(**a**) Schematic illustration of the device-relevant heterolayer structure consisting of ITO/NiO_x_/MAPbI_3_, highlighting the incorporation of a nonionic PTE surfactant within the MAPbI_3_ layer during solution processing. The molecular structure of PTE is shown for reference. (**b**) Photographic images of a bare NiO_x_ film annealed at 300 °C and MAPbI_3_ films fabricated without PTE (pristine MAPbI_3_) and with PTE at concentrations of 30 ppm (MAPbI_3_–PTE30) and 60 ppm (MAPbI_3_–PTE60) on NiO_x_ substrates (1.5 cm × 3.0 cm), demonstrating uniform film formation.

Figure 1a schematically illustrates the fabrication concept and interfacial design strategy employed in this study for MAPbI_3_ photodiodes incorporating PTE on thermally optimized NiO_x_ HTLs. The approach is based on the introduction of a ppm-level polymeric surfactant directly into the MAPbI_3_ precursor solution as opposed to inserting an additional discrete interlayer, thereby enabling the molecular-scale modulation of the NiO_x_/MAPbI_3_ interface during the perovskite film formation.

As shown in Figure 1a, the device-relevant heterostructure consists of an ITO/NiO_x_/MAPbI_3_ stack for which the NiO_x_ HTL annealed at 300 °C provides a Ni^3+^-rich p-type surface with favorable energetics for hole extraction, as described in Section 2.3. During the spin-coating of the MAPbI_3_ precursor containing PTE, the amphiphilic PTE molecules are distributed throughout the wet perovskite film while simultaneously interacting with the underlying NiO_x_ surface. The polar poly(oxyethylene) segments of PTE can coordinate with surface oxygen and nickel sites on NiO_x_, whereas the alkyl chains are oriented toward the perovskite matrix. This configuration is expected to induce an interfacial dipole and modify local nucleation behavior without disrupting the bulk perovskite lattice [36,42,43,44,45,46].

Figure 1b presents photographic images of representative films, specifically a bare NiO_x_ layer annealed at 300 °C and MAPbI_3_ films fabricated without PTE (pristine MAPbI_3_) and with PTE concentrations of 30 ppm (MAPbI_3_–PTE30) and 60 ppm (MAPbI_3_–PTE60). All perovskite films exhibit uniform coverage over a 1.5 cm × 3.0 cm substrate area with no visible macroscopic defects, indicating that the incorporation of PTE does not compromise large-area film formation or processability. The subtle differences in the optical appearance upon the incorporation of PTE suggest changes in microstructural or interfacial properties rather than variations in the film thickness or phase purity.

The fabrication strategy adopted here is specifically designed to decouple bulk perovskite properties from interfacial electronic modification. By introducing PTE at ppm levels into the precursor solution, the intrinsic optical absorption and crystal structure of MAPbI_3_ are preserved, while interfacial interactions at the NiO_x_/MAPbI_3_ interface are selectively tuned. This approach differs fundamentally from conventional interlayer insertion or bulk doping strategies, which often alter charge-transport pathways or introduce additional series resistance [47,48,49].

**Figure A1:**
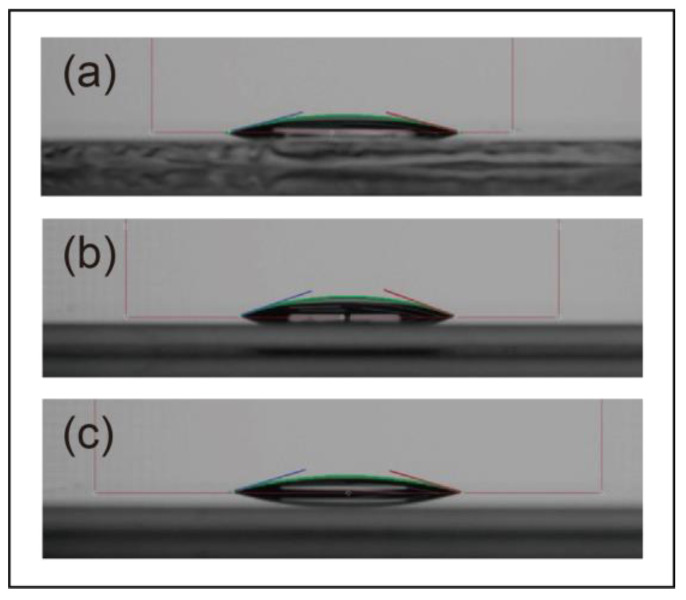
Water-contact-angle images of NiO_x_ films after being coated with DMF/DMSO solutions containing different concentrations of PTE: (**a**) pristine NiO_x_ (0 ppm PTE), (**b**) NiO_x_ with 30 ppm PTE, and (**c**) NiO_x_ with 60 ppm PTE. The increases in the contact angle from 15° (pristine) to 20° (30 ppm) and 17° (60 ppm) indicate adsorption of the amphiphilic polymeric surfactant onto the NiO_x_ surface, resulting in a reduction in the surface free energy and modification of the interfacial polarity prior to perovskite deposition.

To verify that PTE modifies the NiO_x_ surface directly prior to perovskite deposition, independent of perovskite crystallization effects, water-contact-angle measurements of NiO_x_ films coated with DMF/DMSO solutions containing only PTE (without MAPbI_3_ precursors) were taken. Representative droplet images for pristine NiO_x_, NiO_x_ coated with 30 ppm PTE, and NiO_x_ coated with 60 ppm PTE are shown in Figure A1. Pristine NiO_x_ exhibits a highly hydrophilic surface with a contact angle of 15°, consistent with its polar Ni^3+^–O-rich surface. Upon the addition of 30 ppm PTE, the contact angle increases to 20°, while 60 ppm PTE yields a value of 17°, indicating systematic modification of surface wettability by adsorption of the polymeric surfactant.

The increased contact angle reflects a reduction in the surface free energy of NiO_x_ due to the adsorption of amphiphilic PTE molecules. Based on the Young–Dupré relation, the decreases in cos θ from 0.967 (pristine) to 0.942 (30 ppm) and 0.958 (60 ppm) correspond to an interfacial free energy reduction on the order of 2–3%, consistent with the formation of a molecularly thin organic overlayer rather than a thick insulating film [50]. Because these measurements were taken in the absence of MAPbI_3_, they isolate the PTE–NiO_x_ interaction and demonstrate that PTE spontaneously adsorbs and modifies the surface polarity of NiO_x_ prior to perovskite deposition.

The non-monotonic behavior (30 ppm > 60 ppm) further suggests an optimal surface-coverage regime in which PTE forms a molecular-scale, electronically active interfacial layer. This adsorption-induced modification provides a physical basis for the interfacial vacuum-level realignment quantified by subsequent UPS measurements, consistent with established models of polymer-stabilized interfacial dipoles at hybrid oxide/organic interfaces [51].

As will be shown in the following sections, this polymer-mediated interfacial modulation influences (i) perovskite crystallization behavior and grain evolution (Section 3.2), (ii) the bulk optical and structural integrity (Section 3.3), and (iii) the interfacial electronic structure and energy-level alignment (Section 3.4 and Section 3.5). These effects collectively underpin the enhanced carrier extraction, reduced noise, and improved photodetector performance observed at the device level (Section 3.6, Section 3.7 and Section 3.8).

### 3.2. Morphological Evolution of MAPbI_3_ Films Induced by PTE Surfactant Incorporation

**Figure 2:**
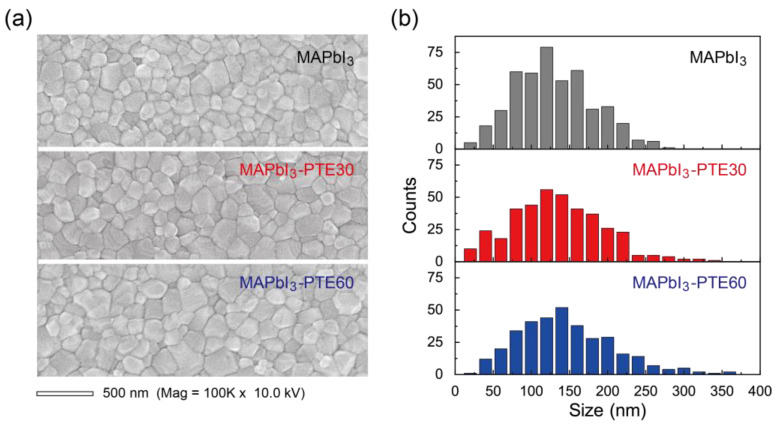
(**a**) Top-view SEM images of MAPbI_3_, MAPbI_3_–PTE30, and MAPbI_3_–PTE60 films deposited onto NiO_x_ HTLs annealed at 300 °C. (**b**) Corresponding grain-size distributions extracted from the SEM images, showing progressive grain enlargement and narrowing of the size distribution with an increase in the PTE concentration.

Based on the fabrication strategy described in Section 3.1, the effects of incorporating PTE on the morphology of MAPbI_3_ films deposited on NiO_x_ HTLs were systematically examined. Given that interfacial crystallization and grain evolution play critical roles in charge transport and recombination processes in perovskite photodiodes, a top-view SEM analysis was conducted to visualize the surface morphology and grain-size distribution of the perovskite layers directly. Figure 2a compares representative SEM images of pristine MAPbI_3_, MAPbI_3_–PTE30, and MAPbI_3_–PTE60 films deposited on NiO_x_ HTLs annealed at 300 °C.

The pristine MAPbI_3_ film exhibits continuous surface coverage with densely packed polycrystalline grains of a moderate lateral size, accompanied by a relatively high density of grain boundaries. Such grain boundaries are known to act as preferential sites for trap-assisted recombinations and ionic migration, which can limit the carrier collection efficiency and increase noise under self-powered operation [31,52,53].

Upon the incorporation of PTE at a concentration of 30 ppm, a pronounced change in the surface morphology is observed. The MAPbI_3_–PTE30 film shows enlarged grains with smoother grain boundaries and a reduced density of small crystallites. This indicates a decrease in the heterogeneous nucleation density during film formation and enhanced lateral grain growth. Increasing the PTE concentration to 60 ppm leads to a more uniform grain structure with a narrower size distribution, suggesting a more homogeneous crystallization process across the film.

The corresponding grain-size distributions extracted from the SEM images are summarized in Figure 2b. A quantitative analysis reveals a progressive increase in the average grain diameter from 119 nm for pristine MAPbI_3_ to 124 nm for MAPbI_3_–PTE30 and 137 nm for MAPbI_3_–PTE60, accompanied by a pronounced extension of the maximum grain size from 272 nm to 330 nm and 415 nm, respectively. This evolution indicates a systematic shift toward larger and more heterogeneous grains with an increase in the PTE concentration, together with clear suppression of the small-grain population. This trend demonstrates that PTE modulates not only the nucleation density but also the lateral grain-growth kinetics during perovskite film formation.

The observed morphological evolution can be rationalized by considering the amphiphilicity of PTE introduced during film formation. As discussed in Section 3.1, PTE molecules distributed within the precursor solution and near the NiO_x_/MAPbI_3_ interface can reduce local surface energy fluctuations and moderate the interaction between the perovskite precursors and the NiO_x_ surface. This effect suppresses excessive heterogeneous nucleation at the interface while promoting controlled crystal growth and grain coalescence [33,37,54]. Importantly, this modulation occurs without introducing a discrete interlayer or altering the bulk composition of MAPbI_3_.

Despite these pronounced changes in the grain morphology, all films remain dense and pinhole-free, indicating that incorporating PTE does not compromise the film continuity or coverage. This is consistent with the uniform macroscopic appearance shown in Figure 1b and confirms that the surfactant-assisted approach preserves processability and scalability.

**Figure A2:**
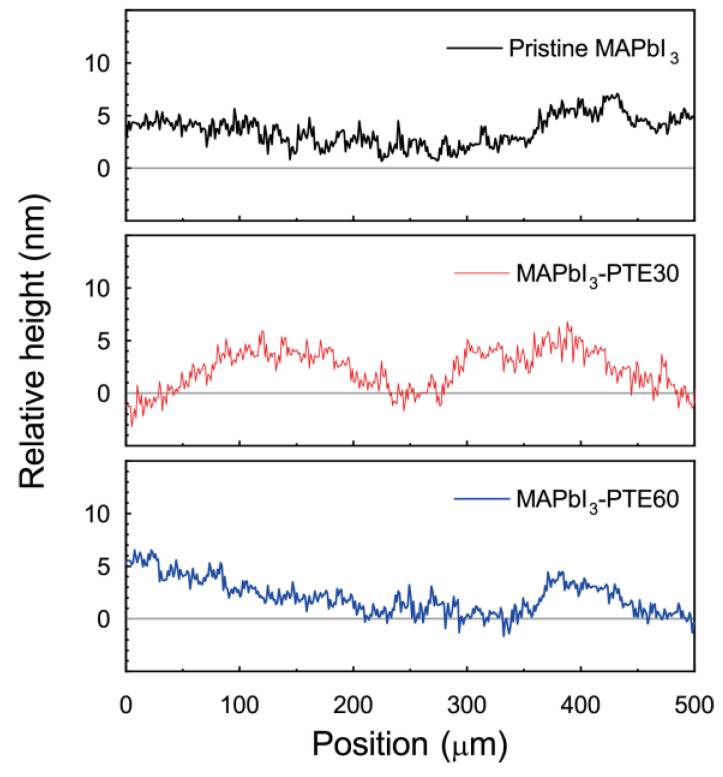
Three-dimensional optical profilometry analysis of perovskite films deposited on NiO_x_ HTLs: (**top**) pristine MAPbI_3_, (**middle**) MAPbI_3_–PTE30, and (**bottom**) MAPbI_3_–PTE60. The corresponding line profiles show comparable surface height variations for all films, yielding RMS roughness values in the approximate range of 2.9–3.4 nm. These results confirm that ppm-level PTE incorporation does not significantly affect vertical film uniformity or surface roughness at interface-relevant length scales, supporting the conclusion that the observed device-performance improvements originate primarily from interfacial electronic modulation rather than morphological smoothing.

For a more in-depth evaluation of whether PTE incorporation affects vertical film uniformity or interface-relevant roughness beyond the lateral grain evolution observed via SEM, three-dimensional (3D) optical profilometry was performed over large-area regions of the perovskite films. Representative surface maps and line profiles for MAPbI_3_, MAPbI_3_–PTE30, and MAPbI_3_–PTE60 films are provided in Figure A2. The root-mean-square (RMS) roughness values were found to be very similar for all samples, within experimental uncertainty levels (approx. 2.9–3.4 nm), indicating that the ppm-level PTE incorporation does not induce additional surface corrugation, thickness non-uniformity, or vertical inhomogeneity at length scales relevant to interfacial charge extraction.

Collectively, the SEM-based lateral grain analysis and large-area 3D profilometry results demonstrate that PTE incorporation primarily modifies the lateral morphology while preserving the vertical film uniformity. The absence of systematic roughness variation indicates that roughness-induced geometric effects are not the dominant origin of the improved device performance. Instead, these results support an interfacial electronic mechanism governed by dipole-induced vacuum-level realignment at the NiO_x_/MAPbI_3_ interface, as elucidated by the UPS analysis (Section 3.4 and Section 3.5) [51,55].

### 3.3. Optical and Structural Integrity of MAPbI_3_ Films Incorporating PTE

**Figure 3:**
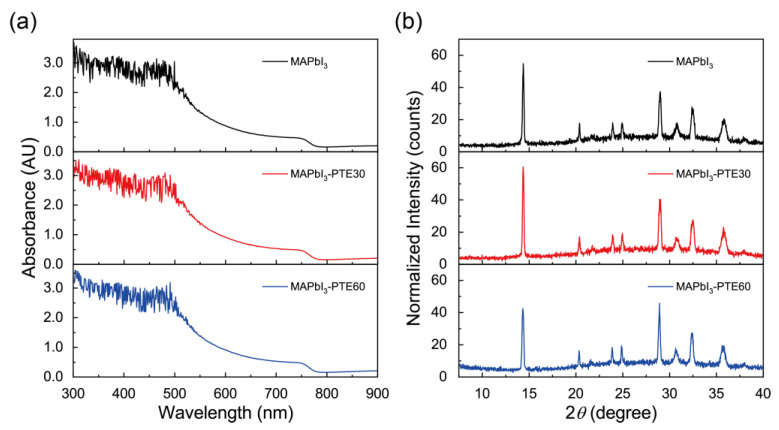
(**a**) UV–vis absorption spectra of MAPbI_3_, MAPbI_3_–PTE30, and MAPbI_3_–PTE60 films on NiO_x_ HTLs. (**b**) XRD patterns of the corresponding films, confirming preservation of the MAPbI_3_ perovskite phase and lattice structure upon PTE incorporation.

To evaluate any potential changes in the bulk optoelectronic properties associated with PTE-induced morphological evolution, the optical absorption capabilities and the crystal structures of the MAPbI_3_ films on the NiO_x_ HTLs were systematically investigated. UV–vis absorption spectroscopy and XRD measurements were performed on pristine MAPbI_3_, MAPbI_3_–PTE30, and MAPbI_3_–PTE60 films, as summarized in Figure 3.

Figure 3a presents the UV–vis absorption spectra of the three films. All samples exhibit strong and broadband absorption across the visible region, extending from approximately 300 to 780 nm, which is characteristic of the intrinsic bandgap of MAPbI_3_. Importantly, the absorption onset, spectral shape, and overall profile remain nearly identical for all PTE concentrations. No discernible shift in the absorption edge or the emergence of additional absorption features could be observed upon PTE incorporation. These results indicate that the optical bandgap and light-harvesting capability of MAPbI_3_ are preserved, and that the incorporation of PTE does not introduce bulk electronic perturbations or secondary phases that affect optical absorption [56].

Any slight differences in the absorption intensity are primarily associated with grain-size-dependent surface morphology effects (Section 3.2) rather than intrinsic changes in the electronic structure. The absence of band-edge modifications confirms that the enhanced device performance reported in later sections does not originate from changes in the bulk absorption or photogeneration efficiency.

The structural properties of the MAPbI_3_ films were more closely examined by XRD, as shown in Figure 3b. All films display sharp diffraction peaks at approximately 14.4°, 28.9°, and 30.7°, corresponding to the (110), (220), and (310) crystallographic planes of the tetragonal MAPbI_3_ phase, respectively [5]. The absence of additional impurity-related peaks confirms high phase purity for all samples regardless of the PTE concentration. Moreover, the peak positions remain unchanged within the experimental resolution, indicating that the lattice parameters and crystal symmetry of MAPbI_3_ are preserved upon PTE incorporation.

While the overall crystal phase and structural integrity remain unchanged, subtle variations in the relative peak intensities are observed among the samples. These variations are consistent with the grain-size enlargement and modified crystallization behavior induced by PTE, as larger and more uniform grains can influence the preferred orientation and diffraction volume without altering the underlying crystal structure [57]. No discernible peak broadening or degradation of the crystallinity is observed, confirming that PTE incorporation does not adversely affect the crystalline quality of MAPbI_3_.

Taken together, the UV–vis and XRD results demonstrate that ppm-level PTE incorporation does not modify the intrinsic optical absorption, band structure, or crystal phase of MAPbI_3_. Instead, PTE influences interfacial interactions and the crystallization kinetics, yielding an improved morphology while preserving the bulk material integrity. This distinction indicates that the performance enhancements discussed below stem from interfacial and morphological optimization rather than bulk electronic modification. Accordingly, the following section directly probes the interfacial electronic structure by UPS (Section 3.4) to elucidate the energy-level alignment and charge-extraction mechanisms.

### 3.4. Interfacial Electronic Structure Modulation Probed by UPS

**Figure 4:**
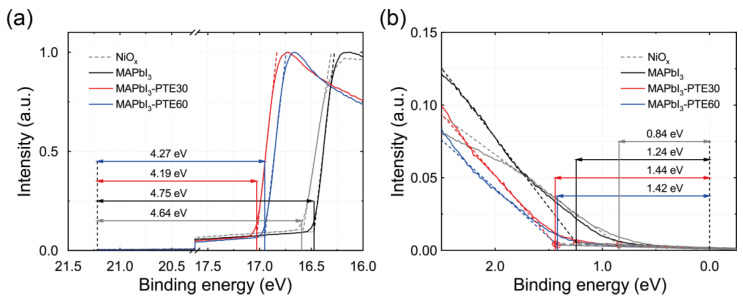
UPS spectra of bare NiO_x_, MAPbI_3_, MAPbI_3_–PTE30, and MAPbI_3_–PTE60 films: (**a**) Secondary electron cutoff regions used to determine the WFs. (**b**) Valence-band regions near *E*_F_ used to extract the VBM positions, illustrating the PTE-induced modulation of vacuum-level alignment at the NiO_x_/MAPbI_3_ interface.

To elucidate the effect of polymer-mediated interfacial engineering on the electronic structure at the NiO_x_/MAPbI_3_ junction more directly, UPS measurements were taken by means of He I (21.22 eV) excitation. UPS spectra were collected for bare NiO_x_ films annealed at 300 °C, pristine MAPbI_3_ films, and MAPbI_3_ films incorporating PTE at concentrations of 30 and 60 ppm (MAPbI_3_–PTE30 and MAPbI_3_–PTE60), as summarized in Figure 4. Because the NiO_x_ HTL was fixed at its thermally optimized condition throughout this study, the UPS-observed changes in WF and apparent valence-band onset can be consistently interpreted as arising from polymer-induced electrostatic vacuum-level shifts (Δ*V*_L_s) rather than from modifications of the intrinsic bulk electronic structure of either NiO_x_ or MAPbI_3_ [51,55].

Figure 4a shows the secondary-electron cutoff regions used to determine the WFs of the respective layers. The thermally optimized NiO_x_ film exhibits a WF of approximately 4.64 eV, consistent with its Ni^3+^-rich p-type electronic characteristic and with previously reported values for sol–gel-derived NiO_x_ annealed at comparable temperatures [26]. Upon the deposition of pristine MAPbI_3_, the WF increases to approximately 4.75 eV, corresponding to a slight vacuum-level step-up (+0.11 eV) relative to NiO_x_.

Notably, the incorporation of PTE induces systematic and concentration-dependent changes in the vacuum-level alignment. For MAPbI_3_–PTE30, the WF decreases to approximately 4.19 eV, while MAPbI_3_–PTE60 exhibits a WF of approximately 4.27 eV. This non-monotonic but clearly PTE-dependent shift in the WF provides direct evidence of the formation of an interfacial dipole layer associated with PTE molecules. The amphiphilic structure of PTE, comprising polar poly(oxyethylene) segments and hydrophobic alkyl chains, enables dipole formation at the NiO_x_/MAPbI_3_ interface via the interaction of the polar moieties with the surface sites of NiO_x_ while the alkyl chains extend toward the perovskite layer. Such dipole-induced Δ*V*_L_ values are a well-established signature of interfacial electronic modulation rather than bulk electronic alterations [45,46,58,59].

Figure 4b displays the valence-band regions near the Fermi level (*E*_F_). The VBM positions were determined relative to *E*_F_ by linear extrapolation of the leading edge of the valence-band spectra, following established UPS analysis procedures. Pristine MAPbI_3_ exhibits a VBM located approximately 1.24 eV below *E*_F_, whereas the MAPbI_3_–PTE30 and MAPbI_3_–PTE60 samples display VBM positions at approximately 1.44 and 1.42 eV below *E*_F_, respectively. In comparison, the VBM of NiO_x_ is located at approximately 0.84 eV below *E*_F_, consistent with its p-type characteristic.

These results indicate that the incorporation of PTE modifies the interfacial band alignment by shifting the effective valence-band energetics of MAPbI_3_ relative to NiO_x_. Notably, this shift originates from vacuum-level modulation associated with interfacial dipole formation rather than from any alteration of the intrinsic electronic structure of the perovskite bulk. This interpretation is fully consistent with the unchanged optical absorption edges and crystal structures confirmed by the UV–vis and XRD analyses (Section 3.3).

It should be emphasized that the UPS trends observed here reflect interfacial electronic tuning confined to the near-surface region as probed by UPS rather than long-range doping or bulk band-structure modifications. In this context, PTE functions as a nonionic polymeric surfactant that physically adsorbs at the NiO_x_/MAPbI_3_ interface and lacks reactive functional groups capable of forming covalent or coordinative bonds with Ni, Pb, or I species. Accordingly, the observed WF changes are attributed to dipole-induced Δ*V*_L_s rather than to chemical bonding or intrinsic band-edge modifications of MAPbI_3_ [51].

Taken together, the UPS results demonstrate that ppm-level incorporation of PTE enables controlled modulation of the vacuum-level alignment at the NiO_x_/MAPbI_3_ interface without perturbing the intrinsic bulk electronic structure of the perovskite. While the valence-band onset of MAPbI_3_ exhibits an apparent shift of approximately 0.18–0.20 eV relative to *E*_F_ upon PTE incorporation, this shift occurs concurrently with an equivalent displacement of the vacuum level, as evidenced by the secondary-electron cutoff. This behavior unambiguously identifies a rigid, dipole-induced Δ*V*_L_ rather than intrinsic band-edge movement or bulk doping of MAPbI_3_.

The magnitude of this Δ*V*_L_ can be quantitatively interpreted using the classical Helmholtz relation, ΔΦ = *N*μ_⊥_/ε_0_, which links the WF change to the interfacial dipole density (*N*) and the perpendicular dipole moment (μ_⊥_) [51,55]. Using literature-reported values of μ_⊥_ for poly(oxyethylene)-based amphiphilic molecules (μ ≈ 2–3 D), the experimentally observed WF reductions of −0.45 eV (PTE30) and −0.37 eV (PTE60) correspond to interfacial dipole densities on the order of 10^13^ cm^−2^.

Importantly, the polymeric attribute of PTE further stabilizes this interfacial dipole through chain entanglement and reduced molecular mobility, distinguishing it from the more transient dipoles typically formed by small-molecule interfacial modifiers [60,61]. As a result, even ppm-level PTE incorporation generates a dense and electronically stable interfacial dipole layer while preserving the MAPbI_3_ bulk band structure, consistent with the unchanged UV–vis absorption and XRD results (Section 3.3).

These UPS-derived Δ*V*_L_ values are explicitly incorporated into the revised energy-level diagrams to clarify how polymer-stabilized dipole formation reduces the hole-extraction barrier (Δ*E*_H_) at the NiO_x_/MAPbI_3_ interface under self-powered operation, thereby enabling enhanced charge extraction, suppressed interfacial recombinations, and improved device performance.

### 3.5. Energy-Level Alignment and Charge-Extraction Mechanism at the NiO_x_/MAPbI_3_ Interface

**Figure 5:**
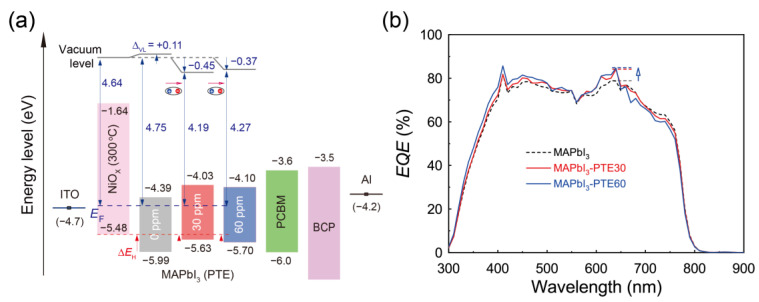
(**a**) Schematic energy-level diagrams of MAPbI_3_ photodiodes fabricated on NiO_x_ HTLs, comparing pristine MAPbI_3_ with MAPbI_3_–PTE30 and MAPbI_3_–PTE60 devices. Energy levels are constructed from UPS-derived values and literature data. The diagram explicitly separates the dipole-induced Δ*V*_L_ from intrinsic band-edge positions, clarifying that the improved hole extraction originates from interfacial energetic realignment without altering the intrinsic band edges of MAPbI_3_. (**b**) EQE spectra of the corresponding devices under zero-bias operation, showing enhanced carrier collection upon PTE incorporation. Arrows indicate the positions of characteristic features discussed in the text.

Building on the UPS analysis in Section 3.4, the role of PTE-mediated interfacial electronic modulation in charge extraction was examined through an energy-level diagram analysis and was correlated with the device photoresponse. Figure 5a presents energy-level diagrams of MAPbI_3_ photodetectors fabricated on NiO_x_ HTLs annealed at 300 °C, comparing pristine and PTE-modified devices. These diagrams explicitly include vacuum-level positions extracted from UPS measurements, allowing the PTE-induced Δ*V*_L_ to be clearly distinguished from intrinsic band-edge positions.

The energy-level diagrams were established using UPS-extracted WFs and VBM positions (Figure 4), together with reported conduction-band levels of MAPbI_3_ and the ETLs [12,26]. Building on the UPS analysis in Section 3.4, the energy-level diagrams in Figure 5a explicitly distinguish dipole-induced Δ*V*_L_ values from the intrinsic band-edge positions of MAPbI_3_. In pristine devices, a finite energetic offset at the NiO_x_/MAPbI_3_ interface forms a barrier for hole extraction under self-powered operation. Upon the incorporation of PTE, the vacuum level undergoes a pronounced downward shift of approximately 0.37–0.45 eV, while the apparent movement of the MAPbI_3_ valence-band onset tracks this displacement, indicating a rigid electrostatic offset rather than intrinsic band-edge reconstruction.

This Δ*V*_L_-driven realignment directly reduces the effective Δ*E*_H_ by approximately the same magnitude and without invoking additional band bending or bulk band-edge reconstruction, consistent with classical interfacial dipole models [51]. Equivalently, the entire perovskite energy manifold is rigidly shifted closer to the NiO_x_ VBM due to vacuum-level realignment rather than through intrinsic modification of the MAPbI_3_ band structure. This dipole-mediated energetic realignment provides a direct physical basis for the enhanced hole extraction and suppressed interfacial recombinations observed in PTE-modified devices.

Upon the incorporation of PTE, the interfacial energy-level alignment is systematically modified. As indicated by the UPS results, PTE induces a Δ*V*_L_ that effectively adjusts the relative position of the MAPbI_3_ VBM with respect to NiO_x_. This modulation reduces the energetic mismatch at the NiO_x_/MAPbI_3_ interface, lowering the effective barrier for hole transfer from the perovskite absorber into the NiO_x_ HTL. Importantly, this effect is achieved without introducing a discrete interlayer or altering the bulk band structure of MAPbI_3_, confirming that PTE functions as an interfacial electronic modifier rather than a dopant.

The physical origin of this energy-level modulation can be attributed to the formation of an interfacial dipole associated with the PTE molecules. The polar poly(oxyethylene) segments of PTE preferentially interact with NiO_x_ surface sites, while the alkyl chains orient toward the MAPbI_3_ layer, establishing an interfacial dipole whose net moment generates the observed Δ*V*_L_. This dipole-driven vacuum-level realignment produces an energetic step-down that lowers the effective Δ*E*_H_, thereby facilitating hole transfer from MAPbI_3_ into NiO_x_ under self-powered operation while preserving the intrinsic electronic structure of the perovskite. This asymmetric energy-level modulation minimizes interfacial carrier accumulation and nonradiative recombinations, providing a direct physical basis for the enhanced hole extraction and suppressed interfacial recombinations which in turn leading to the reduced electronic noise and improved device performance observed in PTE-modified self-powered photodiodes [15].

Although PTE incorporation induces moderate grain-size enlargement (Section 3.2), the dominant origin of the device-performance enhancement arises from interfacial electronic modulation rather than bulk morphological effects. UV–vis absorption and XRD analyses (Section 3.3) confirm that the optical bandgap, crystal phase, and crystallinity of MAPbI_3_ remain essentially unchanged upon the addition of PTE, excluding significant bulk electronic modification.

In contrast, UPS measurements (Section 3.4), together with the revised energy-level diagrams (Figure 5a), reveal pronounced dipole-induced Δ*V*_L_ values at the NiO_x_/MAPbI_3_ interface without measurable intrinsic band-edge movements of MAPbI_3_. This behavior provides direct evidence of polymer-induced interfacial energetic realignment rather than bulk doping or band-structure modifications [51]. The magnitude of this Δ*V*_L_ directly reduces the effective Δ*E*_H_ for hole transport from MAPbI_3_ into NiO_x_ under self-powered operation, establishing a quantitative energetic link between interfacial dipole formation and enhanced device performance.

While enlarged and more uniform grains can reduce grain-boundary trapping and support carrier transport [43,53], such morphological changes alone cannot account for the observed reductions in the extraction barrier, noise spectral density, and recombination losses. Instead, the close quantitative correlation between the PTE-induced Δ*V*_L_ values and the enhancements in the *EQE*, responsivity, and detectivity demonstrates that interfacial energetics, rather than the morphology alone, governs charge extraction and noise suppression in these devices.

With regard to heterojunctions, the dipole-mediated vacuum-level alignment at the NiO_x_/MAPbI_3_ interface is conceptually analogous to the band-engineering strategies widely employed in graphene- and nanowire-based photodetectors, where built-in electric fields and band offsets are designed to promote carrier separation and suppress recombinations under self-powered operation [62,63]. Similarly, graphene/HgCdTe heterojunction infrared photodetectors enhance detectivity through barrier and band-alignment control at the junction interface [64].

In contrast to these structurally complex heterojunction architectures, the present MAPbI_3_ photodiodes achieve effective interfacial energy-level optimization through polymer-stabilized dipole formation induced by ppm-level PTE incorporation during perovskite film formation. As clarified by the UPS-based energy-level diagrams in Figure 5a, PTE incorporation induces a dipole-driven vacuum-level step-down from NiO_x_ to MAPbI_3_, which energetically facilitates hole extraction from MAPbI_3_ toward NiO_x_ under self-powered (zero-bias) operation. This dipole-mediated mechanism reduces the effective Δ*E*_H_ without relying on conventional heterojunction band bending or introducing an additional discrete interlayer. Overall, this comparison underscores the contention that polymer-enabled interfacial dipole engineering provides a fundamentally simpler yet scalable route to interfacial electronic optimization for self-powered photodetectors while retaining performance metrics comparable to those achieved using graphene- or nanowire-based heterojunction platforms.

The consequences of this optimized energy-level alignment are directly manifested in the *EQE* spectra shown in Figure 5b. Compared with pristine MAPbI_3_ devices, both MAPbI_3_–PTE30 and MAPbI_3_–PTE60 exhibit systematically enhanced *EQE* values across the visible spectral range, with the most pronounced improvement observed near 640 nm. At this wavelength, the *EQE* increases from 78.7% for pristine MAPbI_3_ to 84.1% and 84.6% for devices incorporating 30 and 60 ppm PTE, respectively. These enhancements are fully consistent with the suppressed interfacial recombinations and more efficient hole extraction enabled by the improved valence-band alignment at the NiO_x_/MAPbI_3_ interface.

Notably, the overall spectral shape of the *EQE* curves remains unchanged upon PTE incorporation, indicating that the fundamental photo-generation and transport mechanisms are preserved. This enhancement arises primarily from increased carrier collection efficiency rather than changes in the optical absorption or charge-generation pathways. The close similarity between the *EQE* responses of the 30 and 60 ppm PTE devices further suggests that the interfacial modification saturates at low PTE concentrations, consistent with a surface-limited dipole formation mechanism.

Collectively, the energy-level diagrams and *EQE* results reveal that PTE-induced interfacial dipole formation selectively reduces Δ*E*_H_ at the NiO_x_/MAPbI_3_ junction while maintaining balanced carrier transport. This energetically favorable interfacial configuration, enabled by polymer-stabilized dipole-mediated vacuum-level alignment at the NiO_x_/MAPbI_3_ interface, provides a consistent physical origin of the enhanced charge extraction, suppressed recombination, and improved device performance discussed in the subsequent analyses.

It should be noted that this interfacial modulation is concentration-dependent and saturates once sufficient polymer coverage is achieved at the interface. Increasing the polymer content further is therefore not expected to yield additional energetic benefits and may instead limit carrier transport by increasing the effective interfacial thickness or introducing partial insulating capability, as commonly observed in polymer-modified semiconductor interfaces [65]. Accordingly, the present study focuses on the optimal low-ppm regime, while a systematic investigation of effects at higher concentrations will be reported separately.

### 3.6. Current–Voltage Characteristics and Photoresponse Linearity

**Figure 6:**
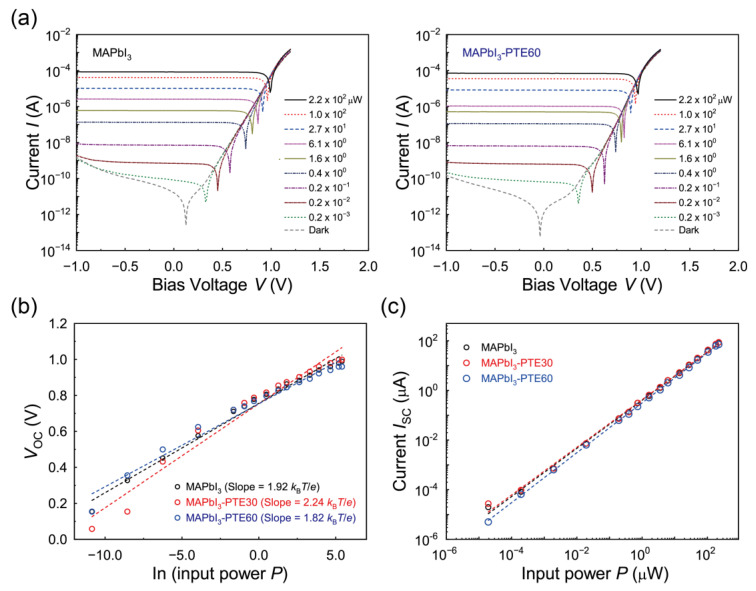
(**a**) Semi-logarithmic *I*–*V* characteristics of MAPbI_3_ (**left**) and MAPbI_3_–PTE60 (**right**) photodiodes measured under 637 nm illumination at varying levels of optical power, together with dark-current curves. (**b**) *V*_OC_ as a function of ln *P* for MAPbI_3_, MAPbI_3_–PTE30, and MAPbI_3_–PTE60 devices. (**c**) *I*_SC_ as a function of *P* on a log–log scale with power-law fitting.

Following the interfacial energy-level optimization discussed in Section 3.5, device-level electrical characteristics were analyzed to assess the effect of PTE-assisted interfacial modulation on the photocarrier dynamics and photoresponse linearity under self-powered operation. Figure 6 shows the *I*–*V* characteristics and light-intensity-dependent photoresponses of MAPbI_3_, MAPbI_3_–PTE30, and MAPbI_3_–PTE60 photodiodes fabricated on NiO_x_ HTLs under 637 nm illumination.

Figure 6a presents representative semi-logarithmic *I*–*V* curves measured over a wide range of incident optical power levels together with the corresponding dark-current characteristics. All devices exhibit clear rectifying diode behavior with a low dark current near zero bias and a systematic increase in the photocurrent with an increase in the illumination intensity. The absence of abnormal leakages or hysteretic behavior indicates stable junction formation and the efficient separation of photogenerated carriers driven by the built-in electric field. Notably, the incorporation of PTE does not alter the overall diode polarity or rectification trend, confirming that the fundamental device architecture and transport pathways remain intact.

To gain insight into the recombination mechanisms and interfacial charge-transport behavior, the open-circuit voltage (*V*_OC_) was analyzed as a function of the natural logarithm of the incident optical power (ln *P*), as shown in Figure 6b. Linear fitting of the *V*_OC_–ln *P* relationship yields slopes corresponding to ideality factors *n* on the order of a unity to two (in units of *k*_B_*T*/*e*), indicating that trap-assisted recombinations remain the dominant recombination pathway for all devices under low-bias operation [66]. The MAPbI_3_–PTE60 device shows a slightly reduced slope compared with pristine MAPbI_3_ that is attributable to the partial mitigation of interfacial recombination losses. This trend is in agreement with the reduced energetic barrier and improved hole-extraction characteristics inferred from the interfacial energy-level alignment discussed in Section 3.5.

Complementary information pertaining to the charge-collection behavior is obtained from the short-circuit current (*I*_SC_) dependence on the incident optical power, as plotted on a log–log scale in Figure 6c. All devices display an approximately linear relationship between *I*_SC_ and *P* over more than five orders of magnitude. Power-law fitting using $I_{SC}\propto P^{\theta }$ yields exponents *θ* close to unity for the MAPbI_3_, MAPbI_3_–PTE30, and MAPbI_3_–PTE60 devices, confirming nearly ideal photodiode behaviors with efficient photocarrier extraction and negligible photoconductive gain or space-charge-limited effects within the investigated power range [15]. The preservation of near-unity *θ* values across all devices indicates that PTE incorporation does not alter the fundamental transport mechanism or introduce nonlinear response artifacts.

Despite the identical power-law exponents, the absolute magnitude of *I*_SC_ is systematically enhanced for PTE-modified devices at a given illumination intensity. The elevated *I*_SC_ levels observed for MAPbI_3_–PTE30 and MAPbI_3_–PTE60 directly reflect more efficient carrier collection under zero-bias conditions, in agreement with the reduced valence-band offset and facilitated hole extraction at the NiO_x_/MAPbI_3_ interface. These results demonstrate that PTE-assisted interfacial engineering increases the collection efficiency without compromising linearity or operational stability.

Based on the *I*_SC_–*P* characteristics in Figure 6c, the zero-bias responsivity at 637 nm was evaluated using *R*_λ_ = *I*_PH_/*P*, where *I*_PH_ = *I*_light_ − *I*_dark_ is the net photocurrent [6,8,10,15,67]. The extracted *R*_637_ values increase monotonically from 406 mA/W for pristine MAPbI_3_ to 434 and 437 mA/W for the MAPbI_3_–PTE30 and MAPbI_3_–PTE60 devices, respectively. This monotonic enhancement corroborates the *EQE*-derived responsivity trends discussed in Section 3.5 and confirms that the improved interfacial energetics translate directly into higher light-to-current conversion efficiencies.

Overall, the combined *I*–*V* characteristics, *V*_OC_–ln *P* analysis findings, and *I*_SC_–*P* scaling results confirm that PTE incorporation at the NiO_x_/MAPbI_3_ interface preserves the fundamental photodiode physics while enabling more efficient charge collection and fewer interfacial recombinations. This electrical optimization directly supports the improved device characteristics and dynamic responses examined in the subsequent sections.

### 3.7. Spectral Responsivity, Noise Characteristics, and Detectivity

Building on the improved charge-collection efficiency and preserved linear photoresponse discussed in Section 3.6, the sensitivity and noise characteristics of the MAPbI_3_ photodetectors were systematically investigated to elucidate the impact of PTE-assisted interfacial engineering on noise-limited performance under self-powered operation.

**Figure 7:**
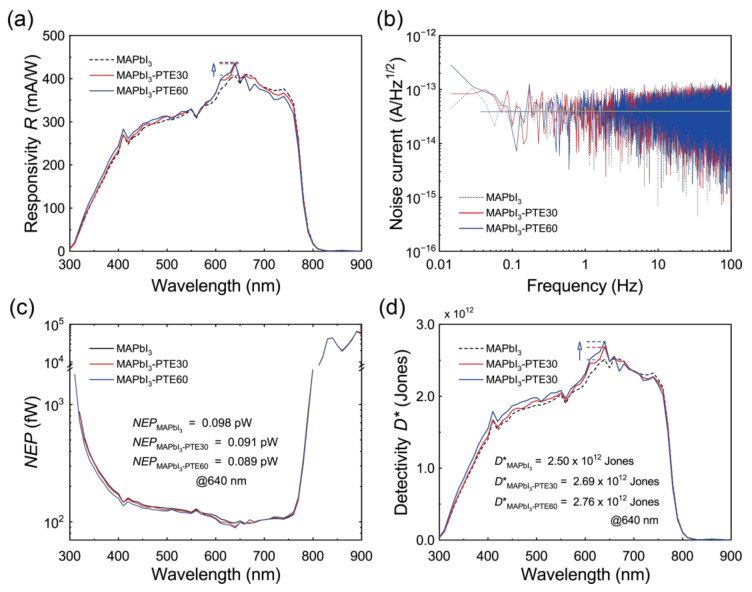
(**a**) Wavelength-dependent responsivity *R_λ_*. (**b**) Frequency-dependent current-noise spectral density *i*_n_. The colored lines indicate the average level of the noise currents. (**c**) *NEP*. (**d**) Spectral specific detectivity D*. All data correspond to MAPbI_3_, MAPbI_3_–PTE30, and MAPbI_3_–PTE60 photodiodes fabricated on NiO_x_ HTLs annealed at 300 °C and measured under zero-bias conditions at room temperature. Arrows indicate the positions of characteristic features discussed in the text.

The wavelength-dependent responsivity *R*_λ_ was derived from the measured *EQE* spectra using the equation $R_{\lambda }=EQE\cdot \frac{e\lambda }{hc}$ [8]. Figure 7a presents the responsivity spectra of the MAPbI_3_, MAPbI_3_–PTE30, and MAPbI_3_–PTE60 devices. All devices exhibit a broadband photoresponse across the visible region with a peak centered near 640 nm, consistent with the absorption profile of MAPbI_3_. Upon the incorporation of PTE, the responsivity increases systematically across the entire spectral range. At 640 nm, *R*_λ_ increases from 406.2 mA/W for pristine MAPbI_3_ to 434.1 and 436.8 mA/W for MAPbI_3_–PTE30 and MAPbI_3_–PTE60, respectively. These values closely match the responsivities independently extracted from the *I*_SC_–*P* analysis (Section 3.6), confirming that the enhanced photocurrent originates from improved charge extraction rather than photoconductive gain.

To evaluate the noise behavior, the current-noise spectral density (*i*_n_) was extracted from dark-current time traces by means of a *FFT* analysis, as shown in Figure 7b [6,7,68,69]. All devices exhibit predominantly frequency-independent noise over the measured range, indicating that white noise dominates under zero-bias operation. Notably, the PTE-modified devices display slightly reduced noise amplitudes (≈3.9 × 10^−14^ A Hz^−1/2^) compared with pristine MAPbI_3_ (≈4.0 × 10^−14^ A Hz^−1/2^), reflecting suppressed electronic noise at the NiO_x_/MAPbI_3_ interface. This trend is consistent with the reduced interfacial recombinations and defect-assisted carrier fluctuations inferred from the *V*_OC_–ln *P* analysis (Section 3.6).

Under self-powered (zero-bias) operation, noise in perovskite photodiodes is predominantly governed by thermal (Johnson) noise and trap-assisted generation–recombination (G–R) noise, while the contribution of shot noise remains negligible due to the extremely low dark current and near-equilibrium junction operation. In such junction-limited devices, interfacial defect states and carrier trapping–detrapping processes act as dominant sources of current fluctuations, particularly at low frequencies [70].

The nearly flat noise spectra presented in Figure 7b indicate that white noise dominates the 1/f noise within the measured frequency window, implying a reduced density of active interfacial fluctuations. Notably, this suppression of electronic noise directly correlates with the reduced *V*_OC_–ln *P* slopes observed for the PTE-modified devices (Section 3.6), providing independent evidence of suppressed trap-assisted recombinations at the NiO_x_/MAPbI_3_ interface.

Together with the dipole-induced vacuum-level realignment revealed by UPS (Section 3.4 and Section 3.5), these results demonstrate that polymer-assisted interfacial dipole stabilization reduces interfacial carrier accumulation and G–R noise, thereby enabling enhanced noise-limited detectivity under self-powered operation. Under zero-bias self-powered operation, noise in perovskite photodiodes is primarily governed by thermal (Johnson) noise and G–R noise, as noted above, whereas pronounced 1/f noise typically arises under biased or photoconductive operation due to trap-assisted carrier capture and release processes [70]. The nearly frequency-independent noise spectral density observed here therefore indicates that the present devices operate in a junction-limited regime within the measured frequency range.

Importantly, this junction-limited noise behavior is consistent with the dipole-induced vacuum-level step-down at the NiO_x_/MAPbI_3_ interface introduced by the incorporation of PTE. As revealed by UPS and illustrated in Figure 5a, the vacuum-level alignment is converted from a slight step-up in the pristine interface to a pronounced step-down upon PTE incorporation, thereby facilitating hole extraction from MAPbI_3_ toward NiO_x_ under self-powered operation. This dipole-driven energetic alignment suppresses interfacial carrier accumulation and reduces the fluctuation pathways associated with trap-assisted recombinations, providing evidence of a direct physical origin of the observed reduction in the noise spectral density. Consistently, the reduced *V*_OC_–ln *P* slopes for the PTE-modified devices (Section 3.6) independently confirm suppressed interfacial recombinations, establishing coherent links among interfacial energetic modulation, recombination kinetics, and noise suppression.

Using the measured responsivity and current-noise spectral density, the noise-equivalent power (*NEP*) was calculated according to *NEP* = *i*_n_/*R*_λ_ [6,7,8,9,10]. As shown in Figure 7c, the *NEP* at 640 nm decreases from 98 fW for pristine MAPbI_3_ to 91 and 89 fW for MAPbI_3_–PTE30 and MAPbI_3_–PTE60, respectively, reflecting the synergistic combination of enhanced responsivity and suppressed noise enabled by PTE-assisted interfacial modulation.

The specific detectivity D* was subsequently evaluated using $D^{*}=\;\sqrt{A\cdot B}/NEP$, where A is the active area and B is the measurement bandwidth (1 Hz) [6,7,8,9,10,15,67,68]. As shown in Figure 7d, the noise-limited detectivity reaches peak values of 2.50 × 10^12^, 2.69 × 10^12^, and 2.76 × 10^12^ Jones at 640 nm for the MAPbI_3_, MAPbI_3_–PTE30, and MAPbI_3_–PTE60 devices, respectively. The systematic increase in D* with the PTE concentration suggests that enhanced carrier collection improves device sensitivity without inducing additional noise. Moreover, the preserved spectral shape of D* indicates that the performance enhancement arises from interfacial electronic effects and not from bulk optical changes.

In addition to detectivity, the *LDR* was evaluated to assess the ability of the devices to maintain a linear photoresponse over a wide range of illumination intensities, a capability critical for imaging and sensing applications. The *LDR* is defined as $LDR=20\log\left(I_{max}/I_{min}\right)$ under noise-limited conditions [6,8,10,15,67]. Notably, the *LDR* values are evaluated under the same zero-bias and 1 Hz bandwidth conditions used for *NEP* and D* extraction, ensuring a consistent noise-limited comparison across devices. Using a maximum incident power $P_{max}$ of 220 µW and the experimentally measured *NEP* values at 640 nm, the *LDR* values were estimated to be approximately 187.0, 187.7, and 187.9 dB for the MAPbI_3_, MAPbI_3_–PTE30, and MAPbI_3_–PTE60 devices, respectively. The consistently high *LDR* across all devices indicates that the incorporation of PTE does not compromise the linear operating range, while the combination of high responsivity and ultralow noise enables an exceptionally broad dynamic range. The ultrabroad *LDR* arises from the combination of near-unity photocurrent scaling exponents and suppressed low-frequency noise, both of which are directly enabled by PTE-mediated interfacial electronic stabilization. This result highlights the advantage of polymer-assisted interfacial control in preserving linear operation over extremely wide dynamic ranges.

When benchmarked against the previously reported self-powered MAPbI_3_ photodiodes summarized in Table 1, the D* values achieved here exceed those of devices employing NiO_x_ HTLs without polymer-induced interfacial dipole modulation and those based on PEDOT:PSS while also being comparable to or slightly higher than state-of-the-art MAPbI_3_ photodiodes incorporating additional interlayers or dopants [12,26,31,32,71,72,73]. Importantly, this competitive noise-limited detectivity is achieved under strict zero-bias operation while maintaining fast response dynamics and an ultrabroad *LDR*.

Although many recent self-powered MAPbI_3_ photodiodes rely on additional interlayers, modified transport layers, or complex heterojunction architectures to enhance sensitivity, the present approach achieves comparable performance through a ppm-level polymeric surfactant directly incorporated into the perovskite precursor. By inducing a dipole-mediated Δ*V*_L_ at the NiO_x_/MAPbI_3_ interface, this strategy reduces the effective Δ*E*_H_ without introducing a discrete interlayer, thereby enabling molecular-scale interfacial modulation with minimal processing complexity. This comparison highlights how polymer-enabled interfacial dipole stabilization provides a simple and scalable route to simultaneously enhance responsivity, suppress electronic noise, and preserve response speeds in self-powered MAPbI_3_ photodiodes.

Beyond perovskite-only comparisons, it is also instructive to benchmark the present devices against graphene- and nanowire-based heterojunction photodetectors reported in the literature. Graphene/ZnO nanowire heterojunction photodetectors have demonstrated detectivity levels on the order of 10^11^–10^12^ Jones under zero or low bias, enabled by band-engineered junctions and built-in electric fields that promote efficient carrier separation and extraction [62,63]. Similarly, graphene/HgCdTe heterojunction infrared photodetectors achieve comparable detectivity levels through barrier and band-alignment control at complex heterointerfaces [64].

In comparison, the MAPbI_3_–PTE60 photodiodes reported here achieve a noise-limited detectivity of approximately 2.8 × 10^12^ Jones under strict zero-bias operation using a significantly simpler, fully solution-processable perovskite diode type of architecture. Rather than relying on band-engineered heterojunctions or multilayer junction stacks, the present approach employs ppm-level polymeric surfactant incorporation to stabilize an interfacial dipole and induce a vacuum-level step-down from NiO_x_ to MAPbI_3_, thereby energetically facilitating hole extraction from MAPbI_3_ toward NiO_x_. This comparison underscores the contention that polymer-enabled interfacial electronic modulation can deliver detectivity levels comparable to those of advanced graphene- or nanowire-based heterojunction photodetectors while offering clear advantages in terms of structural simplicity, processing compatibility, and scalability [51].

In addition to the enhanced sensitivity, preliminary storage-stability tests indicate that the incorporation of PTE contributes to maintaining device stability. After approximately 500 h of ambient storage, the detectivity of all MAPbI_3_ devices decreases by less than ~7%, with no evidence of accelerated degradation induced by the polymer additive. This modest degradation contrasts favorably with PEDOT:PSS-based perovskite optoelectronic devices, which are commonly associated with accelerated performance losses due to moisture ingress and interfacial corrosion [74]. The improved stability observed here is attributed to the chemically inert NiO_x_ HTL combined with a spatially confined, polymer-stabilized interfacial dipole that preserves favorable energetic alignment without introducing hygroscopic or reactive interlayers. Because the devices operate under zero-bias self-powered conditions with an extremely low dark current, this ambient storage stability provides a meaningful first-order indicator of interfacial robustness in the absence of electrical stress. More extended stability studies will be reported in future work.

Collectively, the enhanced responsivity, suppressed noise, elevated detectivity, and ultrabroad *LDR* demonstrate that PTE-assisted interfacial engineering effectively amplifies the intrinsic advantages of thermally optimized NiO_x_ HTLs. This clear correlation between molecular-level interfacial modulation and noise-limited sensitivity motivates the following investigations of the dynamic responses and weak-signal detection performances.

### 3.8. Temporal Response and Frequency-Dependent Detection Performance

Based on the enhanced responsivity, suppressed noise, and elevated detectivity demonstrated in Section 3.7, the dynamic responses of the MAPbI_3_ photodetectors were examined to assess the impact of PTE-assisted interfacial engineering on high-speed, self-powered operation.

**Figure 8:**
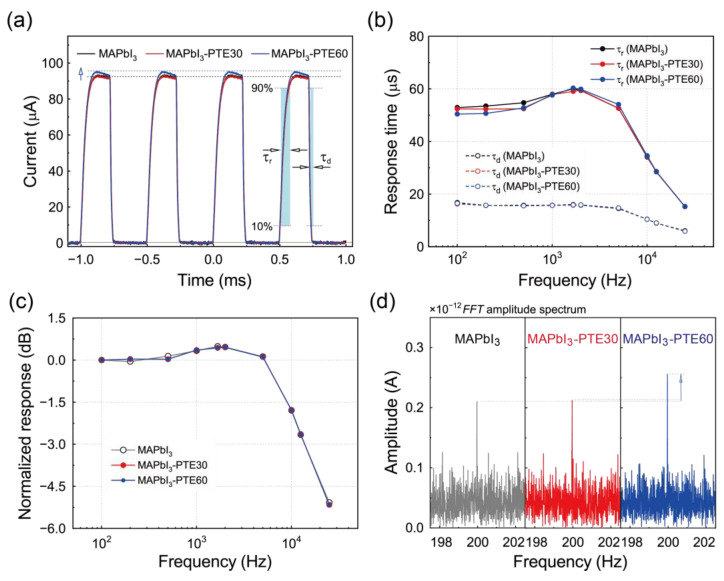
(**a**) Transient photocurrent responses of MAPbI_3_, MAPbI_3_–PTE30, and MAPbI_3_–PTE60 photodiodes measured at zero bias under square-wave-modulated 637 nm illumination (*P* = 220 µW) at 2 kHz. The arrows indicate the positions of characteristic features discussed in the text. (**b**) Extracted rise (τ_r_) and decay (τ_d_) times as a function of the modulation frequency. (**c**) Normalized photocurrent amplitude versus modulation frequency. (**d**) *FFT* amplitude spectra acquired under ultralow-intensity illumination (637 nm, 190 pW) modulated at 200 Hz for the corresponding devices.

Figure 8a shows representative photocurrent transients recorded under square-wave-modulated 637 nm illumination with an incident power of 220 μW at a modulation frequency of 2 kHz. All devices, in this case the MAPbI_3_, MAPbI_3_–PTE30, and MAPbI_3_–PTE60 devices, exhibit sharp and reproducible on/off switching with stable baselines, confirming robust self-powered operation without external bias. Quantitative analyses yield nearly identical corresponding rise times (τ_r_) of 59.4, 59.5, and 59.9 μs and decay times (τ_d_) of 15.8, 15.9, and 15.9 μs. The nearly unchanged τ_r_ and τ_d_ values across all devices confirm that PTE-assisted interfacial modification does not hinder charge transport or induce additional interfacial charging, thereby maintaining fast carrier extraction dynamics.

The frequency dependence of the temporal response is summarized in Figure 8b, where τ_r_ and τ_d_ are plotted as functions of the modulation frequency. For all devices, τ_r_ decreases gradually with an increase in the frequency up to the kilohertz regime, whereas τ_d_ remains nearly constant. The frequency dependence suggests limited modulation-rate sensitivity in the turn-on process, whereas recombination and discharge processes remain stable, demonstrating that PTE-assisted interfacial modification preserves high-speed device operation.

Figure 8c presents the normalized photocurrent amplitude as a function of the modulation frequency. All devices maintain flat normalized responses up to several kilohertz, followed by a gradual roll-off at higher frequencies. The −3 dB cutoff frequencies are located in the range of approximately 10–13 kHz for all three devices, consistent with the sub-100 μs rise and decay times extracted from the transient measurements. While the MAPbI_3_–PTE60 device exhibits slightly higher normalized responses in the intermediate frequency range of approximately 1–5 kHz, the overall bandwidth remains comparable across all devices. These results indicate that the ultimate bandwidth is governed by intrinsic device properties rather than by interfacial modifications.

To evaluate the weak-signal detection in the frequency domain further, the *FFT* amplitude spectra were acquired under ultralow-intensity illumination at 637 nm with an incident power of 190 pW modulated at 200 Hz (Figure 8d). Distinct spectral peaks at the modulation frequency are clearly resolved for all devices, confirming reliable weak-signal detection under zero-bias operation. Notably, the PTE-modified devices exhibit higher *FFT* signal amplitudes compared to those of pristine MAPbI_3_, consistent with their enhanced responsivity and suppressed noise characteristics, as discussed in Section 3.7.

Taken together, the temporal and frequency-domain analyses demonstrate that PTE-assisted interfacial engineering enables MAPbI_3_ photodetectors to achieve simultaneously high sensitivity and fast dynamic responses under self-powered operation. The preservation of rapid rise and decay times, kilohertz-level bandwidths, and reliable weak-signal detection confirms that molecular-level interfacial modulation enhances charge extraction and suppresses noise without compromising response speeds. Combined with the enhanced responsivity and detectivity discussed above, these results establish a coherent framework in which interfacial electronic optimization translates directly into robust, low-noise, and high-speed photodetector performance suitable for practical weak-light sensing and imaging applications.

## 4. Conclusions

In this work, we present a polymer-assisted interfacial engineering strategy that synergistically enhances the performance of self-powered MAPbI_3_ photodiodes through the combination of thermally optimized NiO_x_ HTLs and the ppm-level incorporation of a nonionic polymeric surfactant, PTE, into the perovskite precursor. Systematic structural, spectroscopic, and device-level analyses consistently identify NiO_x_ annealed at 300 °C as a robust baseline that provides favorable energetics for hole extraction, which can be refined further through a molecular-level interfacial modification strategy without introducing an additional discrete interlayer. The incorporation of PTE at ppm-level concentrations selectively modulates the NiO_x_/MAPbI_3_ interface, leading to enlarged and more uniform perovskite grains while preserving the intrinsic optical absorption and crystal structure of MAPbI_3_. UPS reveals that PTE induces an interfacial dipole that induces a rigid Δ*V*_L_ without intrinsic band-edge modification, thereby lowering Δ*E*_H_ under zero-bias operation. Consistent with the UPS analysis, the observed decreases in the WF (from 4.75 eV for pristine MAPbI_3_ to 4.19 and 4.27 eV for PTE30 and PTE60, respectively) originate from dipole-induced vacuum-level realignment rather than bulk electronic reconstruction. This interfacial electronic tuning directly translates into enhanced carrier collection efficiency, as evidenced by an increase in the *EQE* at 640 nm from 78.7% for pristine MAPbI_3_ to 84.6% for PTE-modified devices, accompanied by a corresponding rise in the responsivity from 406 to 437 mA/W. Beyond steady-state performance, PTE-assisted interfacial engineering simultaneously suppresses electronic noise, resulting in reduced *NEP* values and a noise-limited specific detectivity D* of up to 2.76 × 10^12^ Jones under self-powered conditions. Unlike small-molecule surfactants or ionic additives, polymeric surfactants such as PTE provide extended chain conformations, enhanced dipole stability, and suppressed long-range diffusion, enabling robust and spatially confined interfacial electronic modulation. Importantly, these gains in sensitivity are achieved without compromising the fundamental diode operation, photoresponse linearity, or dynamic performance. All devices retain fast temporal responses with τ_r_ and τ_d_ values of approximately 60 and 16 μs, respectively, as well as kilohertz-level bandwidths and reliable weak-signal detection in the frequency domain. The preservation of the response speeds confirms that PTE primarily improves interfacial charge extraction and recombination suppression rather than altering intrinsic transport pathways. A key advantage of this approach lies in its simplicity: all performance enhancements are realized using only ppm-level polymeric additives directly incorporated into the perovskite precursor, without requiring additional interlayers, surface treatments, or complex heterostructure engineering. This molecular-scale interfacial strategy exploits dipole-driven vacuum-level alignment, enabling high performance with minimal material complexity. In addition, preliminary ambient storage tests indicate that the PTE-assisted NiO_x_/MAPbI_3_ interface preserves device stability, with detectivity degradation of less than approximately 7% observed over ≈500 h, demonstrating that the incorporation of the ppm-level polymer does not compromise interfacial robustness under self-powered operation. Taken together, these results demonstrate that polymeric-surfactant-assisted interfacial engineering can synergistically amplify the benefits of thermally optimized NiO_x_ HTLs, enabling self-powered MAPbI_3_ photodiodes that simultaneously achieve high responsivity, suppressed noise, a broad *LDR*, and a fast temporal response. Beyond the demonstrated performance gains, the use of a ppm-level polymeric surfactant directly incorporated into the perovskite precursor offers an intrinsically simple and manufacturing-friendly route to interfacial control. By avoiding additional discrete interlayers, vacuum deposition, or complex heterostructure engineering, this strategy minimizes processing complexity and parasitic resistance while maintaining effective and stable interfacial electronic modulation. Owing to its simplicity and full compatibility with solution processing, this additive-based strategy establishes a practical and scalable design rule for tailoring interfacial energetics in noise-sensitive perovskite photodiodes. By combining molecular-level interfacial dipole stabilization with scalable solution processing, polymer-enabled self-powered perovskite photodiodes emerge as promising building blocks for next-generation low-power optoelectronics, flexible sensing platforms, and renewable-energy-integrated, bias-free photodetector systems. Looking ahead, such polymer-enabled self-powered perovskite photodiodes represent a scalable foundation for low-power optoelectronic components in renewable-energy-integrated systems, where autonomous, bias-free operation and high energy efficiency are critical.

## Figures and Tables

**Table 1:** Comparison of key performance parameters for self-powered MAPbI_3_ photodiodes reported in recent literature (2021–2025) and in this work, with a focus on strict zero-bias operation. *R*_λ_, D*, response speeds, and *LDR*, where available, are summarized to enable fair benchmarking.

Layout	Device Configuration	Wavelength (nm)	*R*_λ_ (mA/W)	D* (Jones)	Rise/Decay Time	LDR	Ref.
-	ITO/NiO_x_/MAPbI_3_:PTE/PCBM/BCP/Ag	640	437	2.76 × 10^12^	60/16 μs (at 2 kHz)	188	This work
-	ITO/NiO_x_/MAPbI_3_/PCBM/BCP/Ag	640	426	2.50 × 10^12^	65/17 μs (at 2 kHz)	187	[26]
-	ITO/NiO_x_/MAPbI_3_/PCBM/ZnO NPs/BCP/Al	594	360	-	0.9/1.8 ms		[12]
	ITO/NiO_x_/MAPbI_3_/PMMA/PCBM/ZnO/BCP/Al	637	401	1.1 × 10^12^	50/17 μs (at 2 kHz)	127	[31]
W/doping	ITO/NiO_x_:PbI_2_/MAPbI_3_/ C_60_/BCP/Ag	-	360	4.0 × 10^12^	-	112	[71]
	ITO/Mg:NiO/MAPbI_3_/C_60_/BCP/Cu	640	410	-	115/11 μs (at 3 kHz)	124	[72]
W/additional layer	ITO/NiO_x_/PMMA/MAPbI_3_/PMMA/PCBM/ZnO/BCP/Al	637	401	1.3 × 10^12^	57/18 μs (at 2 kHz)	139	[32]
	ITO/PEDOT:PSS/PAA-PI/MAPbI_3_/PCBM_60_/ZnO/BCP/Al	637	371	7.82 × 10^10^	61/18 μs (at 2 kHz)	103	[73]

For consistency, detectivity values estimated solely from the dark current are excluded from this comparison.

## Data Availability

The original contributions presented in this study are included in the article. Further inquiries can be directed to the corresponding author.
